# Effects of point-of-care ultrasonography on clinical decisions for suspected hypervolemia: a cross-sectional study of two independent samples

**DOI:** 10.1016/j.clinsp.2026.101052

**Published:** 2026-07-16

**Authors:** Arthur Gus Manfro, Matheus Klauberg, Mateus Correa Lech, Jordano Bandera, Lorenzo Boemler Busato, Vitor Campagnolo, Samuel Boeira, Fernanda Fuzinatto, Dimitris Varvaki Rados, Ana Claudia Tonelli

**Affiliations:** a Internal Medicine Service, Hospital de Clínicas de Porto Alegre (HCPA), Porto Alegre, RS, Brazil; b Internal Medicine Service, Hospital Nossa Senhora da Conceição, Porto Alegre, RS, Brazil; c Faculdade de Medicina, Universidade Federal do Rio Grande do Sul (UFRGS), Porto Alegre, RS, Brazil

**Keywords:** Fluid status, Medical decision, Hospitalized patients, Lung ultrasound, Point-of-care ultrasound

## Abstract

**Background:**

Point-Of-Care Ultrasound (POCUS) usage is rapidly expanding into clinical practice. One of the clinical uses of POCUS is assessing the volume status, considering the limitation of evaluating congestion with the clinical exam.

**Objective:**

Evaluate whether POCUS modified therapeutic plans regarding the patient's volemic status (fluid infusion, diuretic, and vasodilator therapy).

**Methods:**

Cross-sectional observational study performed in hospitalized patients in general clinical wards of two large public teaching hospitals in Brazil (initial and confirmatory sets). Following the request for a POCUS evaluation, physicians were asked if the ultrasound information impacted therapeutic decisions.

**Results:**

A total of 139 POCUS evaluations were included in the initial sample (collected from July 2022 through November 2022) and 158 in the confirmatory sample (collected between August 2023 and January 2024). POCUS evaluations changed the therapeutic conduct in 57% of the assessments (95% CI 48.2‒65.2), mainly due to modification in diuretic prescription. All results were similar and confirmed in the second sample.

**Conclusion:**

This hypothesis-generating study shows that POCUS evaluations may be useful for assessing volume status in hospitalized patients and were associated with changes in reported therapeutic plans. The present findings reinforce that incorporating POCUS into their routines can potentially benefit medical education and clinical practice.

## Highlights

• POCUS evaluations changed the therapeutic conduct in most assessments in this hypothesis-generating study.
• Adopting POCUS teaching and development in ward routines has the potential to have positive impacts.
• These findings are consistent in two large public teaching hospitals.

## Introduction

In recent decades, physicians have integrated Point-Of-Care Ultrasound (POCUS) protocols into clinical practice.^1^ Designed to complement traditional physical examinations, POCUS involves the use of ultrasound technology at the patient's bedside by physicians (non-radiologists) directly involved in patient care, aiming to enhance clinical reasoning and decision-making. During this examination, the attending physician acquires and interprets images within the clinical context, providing valuable data that can influence the established care plan.^2^ The medical literature has extensively discussed the utility of POCUS across various domains, encompassing, among others, the assessment of trauma,^3^ the management of mechanical ventilation,^4^ guidance in volume resuscitation,^5^ and fluid status assessment.^6^

Assessment of volume status is crucial for various clinical conditions demanding hospitalization, particularly in cases of decompensated heart failure.^7^ It plays a central role in monitoring patients undergoing high fluid loads (e.g., volume resuscitation, maintenance fluids, perioperative period, renal failure).^8^ In conditions directly linked to congestive states, evaluating fluid status becomes especially important in the routine care of wards, particularly given the limitations of physical examination in assessing residual systemic and pulmonary congestion.^9^ For instance, in heart failure, current evidence demonstrates the significance of POCUS in identifying patients with residual congestion - those exhibiting ultrasonographic signs of pulmonary edema face an elevated risk of rehospitalization and adverse events.^10^^,^^11^ In these clinical scenarios, initial findings or impressions from the physical examination can be modified or confirmed through focused ultrasound evaluation.

The implementation of POCUS for fluid status assessment by trained physicians has the potential to drive adjustments in bedside decision-making. Previous literature has identified that POCUS incorporation may lead to a more rational use of complementary imaging studies and potentially improve clinical outcomes.^12^^,^^13^ Consequently, there is a growing consensus which advocate for the incorporation of POCUS knowledge into formal clinical medical training.^14^^,^^15^ With the results of this study, the authors expect to add to the increasing literature showing the utility of POCUS. The authors aim to evaluate and estimate the magnitude of the impact of POCUS volemic status evaluation performed by in-training professionals on the most basic aspect of clinical care (i.e., change in clinical decision-making).

## Methods and materials

### Study design and setting

This is a cross-sectional study with prospectively collected data that follows STROBE guidelines.^16^ The study was conducted within the clinical teaching wards of two preeminent teaching hospitals in south Brazil (Hospital de Clínicas de Porto Alegre and Hospital Nossa Senhora da Conceição) ‒ collectively housing approximately 1500 beds. These hospitals feature two analogous, yet autonomous, rotating medical teams dedicated to performing POCUS evaluations for inpatients in clinical wards (outside the emergency department and intensive care units). Each team consists of two to three second-year Internal Medicine residents in training alongside two to three expert POCUS supervisors. Most examinations were conducted by second-year residents, classified as intermediate learners in POCUS, under the direct supervision of senior staff with expertise in POCUS (ACT, FF and DVR). In certain instances, senior staff members performed the evaluations indirectly. A POCUS expert was defined as an individual who regularly utilizes POCUS in clinical practice, teaches POCUS courses at local or national levels, and meets at least one of the following criteria: has completed a dedicated POCUS fellowship, holds a leadership position in a national professional society related to POCUS, or has published previously on topics related to POCUS. Moreover, the evaluation of trainees’ skills followed a structured checklist.^17^ In these hospitals, all residents and permanent staff can request POCUS evaluation using a standard online formulary.

### Research sample, inclusion, and exclusion criteria

The study's dataset consisted of two distinct yet comparably structured groups of ultrasound assessments, aiming to maintain consistency in methodology while allowing for comparative analysis across different timeframes and settings. The initial dataset was gathered at Hospital de Clínicas de Porto Alegre between July and November 2022. The study excluded individuals under 18-years of age, cases where POCUS could not be performed due to adverse conditions, and instances where either the patient or the attending physician declined participation. The second dataset, serving as a confirmatory set, was collected at Hospital Nossa Senhora da Conceição from August 2023 to January 2024, when the Internal Medicine department incorporated a POCUS rotation for its residents, adhering to the identical inclusion and exclusion criteria established for the initial dataset. Both datasets were gathered independently but followed a uniform protocol and similar procedural guidelines. Differences between the two sets are delineated in subsequent sections of this paper; where not explicitly mentioned, it is to be understood that the methodologies were mirrored.

### Point of care ultrasonography evaluation protocol and results

The evaluation protocol comprised Lung Ultrasonography (LUS) and Inferior Vena Cava Evaluation (IVC) with patients in a semi-recumbent (30°) position. For LUS, a convex transducer was utilized to assess four specific regions in each hemithorax (anterior-superior, anterior-inferior, lateral-superior, and lateral-inferior). In each region, the field (one intercostal space) was considered positive if it identified the presence of three or more B-lines (hyperechoic vertical artifacts arising from the pleural line following the respiratory cycle and reaching a depth of at least 15 cm without fading). Additionally, in lateral-inferior sites, the ultrasound protocol assessed the presence of pleural effusion (considered present if any of the following was identified: anechoic collection with distance between the inner chest wall and lung was ≥5 mm, lack of mirror image from liver or spleen with positive spine sign). The LUS was deemed compatible with pulmonary congestion if positive regions or pleural effusion were bilaterally present.^16^^,^^18^

The IVC evaluation protocol utilized a phased-array transducer in the subxiphoid window to measure the longitudinal IVC (marker cephalad) and its variation during a spontaneous respiratory cycle. Examiners measured the IVC 1‒2 cm distally to the hepatic vein, grading its size and variability on a standardized scale (Grade 1 = diameter < 21 mm and respiratory variability ≥ 50%; Grade 2 = diameter < 21 mm and respiratory variability < 50% or diameter ≥ 21 mm and respiratory variability ≥ 50%; Grade 3 = diameter ≥ 21 mm and respiratory variability < 50%). Patients with Grade 1 were considered to have a Central Venous Pressure (CVP) of 0‒5 mmHg (mean 3 mmHg), Grade 2 with CVP estimated at 5‒10 mmHg (mean 8 mmHg), and Grade 3 with CVP 10‒20 mmHg (mean 15 mmHg).^19^ The examiners assessed patients using Sonosite M-Turbo®, SonoSite Edge II®, or General Electric LOGIC e R7® ultrasound machines.

In the first week of the POCUS rotation, all exams were performed together by the internal medicine resident, alongside the POCUS preceptor, in order to ensure adequate image acquisition and correct interpretation of ultrasound findings. In most of the following weeks of the rotation, the resident could perform solo examinations; nonetheless, whenever there were doubts regarding the interpretation of the exam or if an image was considered to be of insufficient quality, the resident discussed the images with the preceptor and, if needed, repeated the protocol under supervision.

After performing the evaluation, POCUS teams informed the requesting physician of the evaluation for the requesting physician, both directly (direct contact, telephone, or instant message) and in the electronic medical records. The results included the findings (i.e., presence or absence of B-lines, IVC assessment) and interpretation (compatible with lung congestion, isolated IVC dilatation, LUS indicating other disease, or no alteration). The clinical decision based on these results was at the discretion of the attending physician; no direct therapeutic recommendations were made.

### Data sources, variables, and outcomes

Data collection was performed through online forms. Physicians who requested a POCUS evaluation and agreed to participate in the study were prompted to assess and document their clinical observations about the patient's overall volume status and therapeutic intentions concerning fluid administration, the use of diuretics, and the prescription of vasodilators. In the initial sample, for each treatment option, physicians were required to record their initial decision (prior to POCUS) and their final decision (following POCUS), with options being to initiate/increase, suspend/reduce, or maintain the existing course of treatment. Patient outcomes or prescriptions were not evaluated directly by the research team. Furthermore, within the initial sample, physicians rated on a Likert scale the extent to which the POCUS examination influenced their clinical decision-making. This scale ranged from 1 (not helpful) to 5 (very helpful), providing a quantifiable measure of POCUS’s impact on clinical assessments and therapeutic planning.

### Statistical analyses and sample size

To assess the impact of POCUS evaluations on modifications in therapeutic strategies, the authors determined the prevalence of such changes and their 95% Confidence Intervals (95% CI). The relationship between therapeutic decisions and the physicians' level of experience was analyzed using the chi-square test, and odds ratios were reported. Physicians' experience was grouped as follows: internal medicine residents comprised one group, and attendings and clinical specialties residents (all of whom had already completed internal medicine training) comprised the other. All statistical analyses were executed using IBM SPSS Statistics software, version 25, adopting a significance level of 0.05 for all analyses.

Drawing on existing literature, the objective was to detect alterations in therapeutic decisions following ultrasonographic assessments, with an anticipated change rate of 30%.^20^ To achieve a 95% confidence interval while maintaining a margin of error at 5%, the authors determined that the initial sample size should consist of 323 assessments. In the confirmatory analysis, a sample of 164 evaluations was estimated, considering a 15% margin of error and a 40% therapeutic change.

### Role of the funding source and data availability

This study was funded through the *Research and Events Support Fund* from the same hospital. The funder did not participate in study conduct, analysis, or manuscript writing. Data are stored with the authors, and they will be shared anonymously upon reasonable request.

### Ethics statement

The Ethics Committee of Hospital de Clínicas de Porto Alegre (REB number: 60039722.1.0000.5227) and Hospital Nossa Senhora da Conceição (REDB number: 23.173) approved this research following national guidelines and regulations. All requesting physicians and analyzed patients signed informed consent. Requesting physicians and patients were not coerced in any form, since clinical ward POCUS routine was performed independently of study inclusion.

## Results

### Initial sample ‒ hospital de clínicas de porto alegre

#### Sample description

During the initial sample collection, 165 POCUS assessments for volemic evaluation were performed. There were 25 exclusions as the requesting physician did not complete the questionnaire, and one additional patient refused participation. The authors included 139 POCUS assessments from this total, comprising 113 patients. Most patients were older and self-declared white. Exams were requested mainly by internal medicine residents. Sample description is depicted in Table 1.

**Table 1:** Sample description; data presented in mean ± side deviation.

	Initial sample	Confirmatory sample
	**n = 139 evaluations / 113 patients**	**n = 158 evaluations / 131 patients**
**Age (of individual patients)**	64.9 (±14.5)	
**Sex (male) (of individual patients)**	56 (49.6%)	
**Ethnicity (white) (of individual patients)**	95 (84.0%)	
**Physician requesting the evaluation**		
Internal medicine residents	94 (57.2%)	138 (87.3%)
Clinical specialties residents and attendings	45 (32.3%)	20 (12.7%).

#### Therapeutic decisions

**Fig. 1:**
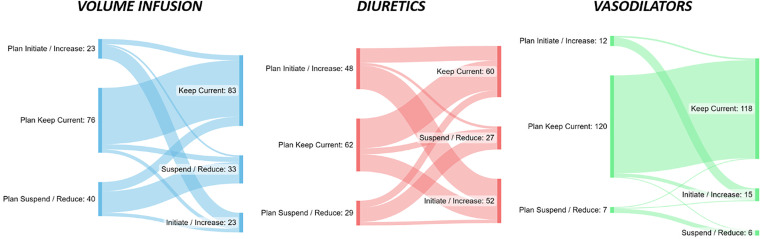
Changes in therapeutic decisions with point-of-care ultrasound information. The original therapeutic plan for each assessment (volume, diuretics and vasodilators) is described on the left, final decisions are described on the right.

Across 139 POCUS assessments, changes in therapeutic decisions were observed in 56.5% of cases (79/139 evaluations, 95% CI 48.2‒65.2). Specifically, fluid infusion plans were modified in 23.7% of evaluations (33/139, 95% CI 16.9‒31.7), diuretic therapy adjustments were made in 42.4% of cases (59/139, 95% CI 34.1‒51.1), and vasodilator administration was altered in 6.5% of instances (9/139, 95% CI 3.0‒11.9). These results are depicted in Fig. 1.

The overall change in therapeutic decisions was not significantly associated with physician experience (OR = 0.543; 95% CI 0.265‒1.114). However, upon analyzing individual components of the therapeutic plan, it was found that more experienced physicians (clinical specialty residents or attendings) were less inclined to alter volume infusion plans post-POCUS (OR = 0.295; 95% CI 0.105‒0.825). Nevertheless, there was no significant association observed for diuretics (OR = 0.862; 95% CI 0.418‒1.775) or vasodilators (OR = 0.578; 95% CI 0.115 ‒ ∞).

### Confirmatory sample ‒ hospital Nossa Senhora da Conceição

In the confirmatory sample, a total of 164 POCUS assessments were initially considered for eligibility. There were six exclusions due to inadequate IVC visualization, resulting in 158 assessments included in the final analysis, encompassing 131 individual patients. Regarding therapeutic decisions, results indicated that 54.4% of the evaluations led to changes in previous treatment plans (86/158 evaluations, 95% CI 46.5%‒62.2%).

## Discussion

In this cross-sectional descriptive study, the authors observed that the incorporation of POCUS data prompted management changes in over 50% of cases across two distinct samples, surpassing prior estimates found in the literature, which suggested a change rate of around 30%.^20^, ^21^, ^22^ These results must be interpreted as possible positive impacts of incorporating POCUS into ward routines, but definitive conclusions cannot be drawn directly by this study, considering its cross-sectional design. Thus, this study should be considered primarily and hypothesis-generating. Previous literature findings have already described POCUS sensitivity to identify congestion states superior to isolated clinical examination^23^ and highlighted how attending physicians reassess patient volume status following POCUS evaluations.^24^ The present study contributes to this body of knowledge by demonstrating that changes in management decisions within a tertiary teaching hospital setting may be more pronounced than previously recognized. Interestingly, the authors observe little correlation between clinical experience and the propensity to alter management decisions based on POCUS findings – with the caveat that subanalyses of this sample are underpowered to draw definitive conclusions.

Positive outcomes and pearls associated with POCUS incorporation over the past decade have led to a growing recognition of the importance of integrating adequate bedside ultrasonographic assessments into clinical practice and medical education.^25^^,^^26^ In light of this, proficiency in POCUS is increasingly considered essential not only for medical residents^15^ but also for undergraduate students in medical training.^27^ Furthermore, it has the potential to yield economic benefits for the healthcare system by reducing costs associated with additional diagnostic tests.^28^ Moreover, ultrasound protocols, particularly those that are highly trainable and rapid to perform, such as LUS, have been compared favorably to traditional physical examination methods, radiological strategies, and laboratory tests, demonstrating the capacity to detect clinically significant problems effectively.^29^^,^^30^ Recognizing this potential, the American College of Physicians has issued a statement acknowledging the pivotal role of POCUS in internal medicine and encouraging physicians and physicians-in-training to acquire proficiency in this skill set.^31^ Recent editorials have challenged traditional views of clinical examination, referring to POCUS as a potential fifth pillar in the physical examination, alongside inspection, palpation, percussion, and auscultation.^32^ The exploratory and hypothesis-generating study is potentially in agreement with the POCUS pearls, demonstrating a possible positive impact on conduct arrangement.

However, pitfalls from POCUS assessments must also be acknowledged: 1) False-positive findings may occur due to artifacts, suboptimal image acquisition, or over-interpretation of nonspecific signs, potentially leading to unnecessary interventions; 2) Diagnostic accuracy is strongly operator-dependent and influenced by the learning curve; 3) POCUS findings are highly context-dependent and should always be interpreted in conjunction with clinical presentation, pre-test probability, and eventual complementary investigations rather than in isolation.^32^^,^^33^ It is likely that some of those pitfalls may have influenced part of these findings, as they would in the incorporation of any new technology in clinical wards and medical training curriculum.

The present study has several strengths. Firstly, both samples were sourced from large teaching hospitals, where medical residents and staff routinely request POCUS examinations to inform clinical decision-making and administer treatments accordingly. This ensures that this data is grounded in real-world clinical practice, reflecting the complexities and nuances encountered in everyday patient care scenarios. Even considering that POCUS pitfalls may have influenced some of these findings, this should be interpreted as an impact of its integration into clinical practice. Furthermore, the authors confirmed the reliability of these results by validating the findings of the initial sample through an independent additional set of patients. This confirmatory analysis strengthens the findings of the initial sample and reinforces external validity in teaching hospitals with structured POCUS programs. Nevertheless, the validity of these findings in other settings (community hospitals or different patient populations) cannot be fully validated by the current study.

The present study also has significant limitations. Firstly, the authors did not achieve the intended sample size, potentially introducing increased statistical uncertainty in the sample. Even though the significant impact observed on therapeutic decisions and replication of findings in a second independent sample may mitigate this limitation to some extent, caution should be placed when interpreting these results, since neither of the samples achieved the targeted number for the study population. For the sensitivity/subgroup analyses, this limitation is even more worrisome – since those analyses are likely underpowered. The cross-sectional nature of the study and lack of blinding may introduce social desirability biases, rendering the study results as hypothesis-generating rather than definitive conclusions. Also, the authors unfortunately were unable to analyze both samples together due to the absence of certain epidemiological data, management change types, and specific physical examination data in the second sample. Secondly, a very relevant limitation of this methodology was the lack of direct assessment of whether the reported treatment plans were implemented in patients' prescriptions. This could confirm if the reported decisions were in fact implemented and would serve as a reference standard for POCUS examination and a confirmation of these findings. If available, this data would also provide valuable insights into the practical application of the present findings. Thirdly, the absence of direct measurement of clinical outcomes, such as mortality, rehospitalization rates, or length of hospital stay, limits the authors’ ability to evaluate the tangible impact of POCUS-guided management on patient outcomes. The lack of baseline characteristics and measures of the patients precludes sensitivity analyses correcting for possible interference of potential confounders (e.g., reason for hospital admission, patient comorbidities and baseline therapy). However, since soliciting physicians had access to full patient information, it is reasonable to understand that conduct modification was implemented considering these undescribed factors. Fourthly, the authors used a simplified protocol based on the CATUS assessment, which excluded direct cardiac evaluation ‒ this can impact in not recognizing more subtle congestion states, which may have been labeled as “inconclusive”. The incorporation of more advanced POCUS techniques, such as direct echocardiography and E/e’ measurement, could potentially modify the reported results. Lastly, despite close supervision from trained professionals, a significant proportion of POCUS examinations were conducted by medical residents, and images were not evaluated in duplicate. Consequently, there exists a risk of erroneous interpretation of POCUS findings, which could potentially influence the accuracy and reliability of these results.

The present study explores data from two teaching hospitals, revealing that ultrasonography examination led to changes in the initial therapeutic plan in more than half of the patients. These findings agree with the modern tendency to consider POCUS as a potential transformative tool with a significant impact on patient care, enhancing the diagnostic and decision-making process in clinical practice. The present study shows that POCUS training programs can be successfully integrated into ward routines in teaching hospitals and that trainees can acquire skills to perform assessments capable of helping requesting physicians to reevaluate therapeutic plans. However, the cross-sectional and unblinded nature of the study warrants caution in interpreting such results as definitive, since further studies should focus on hard clinical outcomes rather than surrogate outcomes ‒ such as duration of hospitalization, failure of decongestion, re-hospitalization, and mortality – preferably and prospective methodology. Additional studies may also be conducted to verify POCUS's capacity to enhance clinical examination skills. In conclusion, these findings emphasize that adopting POCUS teaching and development in ward routines has the potential to influence and prompt modifications in therapeutic decisions regarding volume status. This underscores the importance of integrating POCUS training into medical education curricula to equip future healthcare providers with the skills necessary to leverage this diagnostic modality in patient care.

## Authorship declaration

The authors declare that the authorship listing adheres to the journal's policy and that all authors agree with the content of the submitted manuscript.

## Authors’ contributions

AGM contributed to conceptualization, data curation, formal analysis, writing-original draft preparation, writing-review & editing; MK and ML contributed to conceptualization, data curation, formal analysis, writing-original draft preparation; JB, LBB, VC, SB, and FF contributed to writing-original draft preparation and data acquisition; DVR contributed to supervision, writing-review & editing; ACT contributed to conceptualization and ideation, methodology, writing-review & editing.

## Funding

Research and Event Support Fund from Hospital de Clínicas de Porto Alegre.

## Data availability statement

The datasets generated and/or analyzed during the current study are available from the corresponding author upon reasonable request.

## Declaration of competing interest

The authors declare no conflicts of interest.
